# Association of vitamin D with HIV infected individuals, TB infected individuals, and HIV-TB co-infected individuals: a systematic review and meta-analysis

**DOI:** 10.3389/fpubh.2024.1344024

**Published:** 2024-02-14

**Authors:** Kaidi Xie, Yang Zhang, Mei Zhang, Hao Wu, Luyao Zheng, Jiahao Ji, Zhen Li, Wen Wang, Tong Zhang

**Affiliations:** ^1^Center for Infectious Diseases, Beijing Youan Hospital, Capital Medical University, Beijing, China; ^2^Beijing Key Laboratory for HIV/AIDS Research, Beijing, China

**Keywords:** HIV, TB, HIV-TB, vitamin D deficiency, prevalence, supplementation vitamin D

## Abstract

**Background:**

Vitamin D deficiency (VDD) is a worldwide disease. VDD is also associated with an increased risk of HIV-related comorbidities and mortality, and patients have a tendency to develop active tuberculosis compared to those with latent tuberculosis infection. Vitamin D supplementation may modulate HIV replication, improve TB inflammation and reduce progression of HIV-TB co-infection.

**Methods:**

We meta-analyzed individual participant data from cohort studies, cross-sectional study, and RCTs of vitamin D in HIV group, TB group, and HIV-TB group. The primary outcomes were differences in vitamin D level and VDD prevalence between three groups, the secondary outcomes were CD4 count, HIV viral load, time to sputum smear conversion, time to culture conversion, relapse, morality, and TB score.

**Results:**

For vitamin D levels, the overall mean difference (MD) between HIV group and TB group was −0.21 (95% CI, −20.80–20.38; *p* = 0.9, *I*^2^ = 84%), HIV group and HIV-TB group was 0.87 (95% CI, −11.45–13.20; *p* = 0.89, *I*^2^ = 87%), and TB group and HIV-TB group was 1.17 (95% CI, −5.21–7.55; *p* = 0.72, *I*^2^ = 85%). For vitamin D deficiency prevalence, the overall odds ratio (OR) for HIV group versus TB group was 1.23 (95% CI, 0.46–3.31; *p* = 0.68; *I*^2^ = 70%), HIV group versus HIV-TB group was 1.53 (95% CI, 1.03–2.29; *p* = 0.04; *I*^2^ = 0%), and TB group versus HIV-TB group was 0.85 (95% CI, 0.61–1.20; *p* = 0.36; *I*^2^ = 22%). In HIV-TB group, the overall OR for vitamin D group versus placebo group was 0.78 (95% CI, 0.34–1.67; *p* = 0.52; *I*^2^ = 60%).

**Conclusion:**

Our findings indicated that there were no variations in vitamin D levels between three groups. The prevalence of vitamin D deficiency was higher in the HIV-TB group than in the HIV group. Additionally, the administration of vitamin D supplements did not have obvious impact on CD4 count and viral load. Likewise, vitamin D had no effect on time to sputum smear conversion, time to culture conversion, relapse, 12-month morality, and TB score.

## 1 Introduction

Vitamin D deficiency (VDD) is a worldwide disorder, with a high prevalence in the general population of both Western and developing countries (1). Approximately 7% of the population presents with <30 ng/mL of serum vitamin D concentrations worldwide (2). VDD may cause immune dysfunction by altering the expression of autophagy and inflammatory markers in HIV-infected patients. A systematic review showed that HIV infected subjects were prone to have VDD compared with general population. ART, older age, lower BMI, lower latitude, and male sex may present risk factors for VDD in PLWH (3). Additionally, its deficiency is also linked with an increased risk of AIDS-related comorbidities and mortalities (4, 5). Huang et al. explored the VDD was associated with an increased risk of developing active tuberculosis in subjects with latent tuberculosis infection (6, 7) and was associated with an increased risk of tuberculin skin test conversion/tuberculosis infection conversion, and there was a trend for subjects with active tuberculosis to have lower levels of vitamin D compared to those with latent tuberculosis infection did not reach statistical significance, suggesting that VDD is more likely to be a risk factor rather than a consequence of tuberculosis disease (6). A systematic review showed that up to 88.9% of TB patients had VDD, with the main predictors being lack of ultraviolet exposure, inadequate dietary intake, comorbidities, and old age (8). For HIV-TB co-infected patients, VDD at ART initiation were independently associated with increased risk of incident TB (9).

Vitamin D is involved in many aspects of the body’s metabolism and functioning, regulating calcium levels, parathyroid hormone and calcitonin production, bone mineral density, as well as innate immunity (10), inflammation (11), respiratory infection prevention (12), pregnancy (13) and thyroid dysfunction (14). The ability of vitamin D to control infections and the autoimmune system is becoming a new idea in disease treatment (15).

Vitamin D has attracted interest as a potential drug candidate with its historical use in TB treatment (16). In addition, HIV transcription will be regulated by vitamin D supplementation. The regulation of cytokines and chemokines by vitamin D has implications not only for inflammation in TB, but also for HIV replication (17). Vitamin D did not influence time to sputum culture conversion overall, but it accelerated sputum culture conversion in patients with multidrug-resistant pulmonary TB (18). Akimbekov et al. reported that 4 mg of zoledronic acid per year, supplemented with 400 mg/day of elemental calcium and 1.25 mg/month of vitamin D3, is a potent and effective treatment for osteopenia and osteoporosis in HIV-infected patients (19). In another study, Huang et al. also confirmed that annual dosing of 5 mg zoledronate, following 12 months daily 1 g calcium and 50,000 IU vitamin D supplements treat bone loss in HIV-infected patients (20). Its supplementation reduces the coinfection and progression of HIV/TB (17).

Previous studies have generally analyzed limited groups, did not all involve three groups (HIV group, TB group, and HIV-TB group). Moreover, the primary results and secondary results of the previous studies were not comprehensive enough, and they did not all involve differences in vitamin D levels among the three groups, differences in the prevalence of VDD, as well as the impact of vitamin D supplementation on patient mortality, sputum smear conversion time, sputum culture conversion time, CD4 cell count, and the impact of HIV viral load, relapse, and TB score. Thus, this meta-analysis aimed to evaluate the differences in vitamin D levels and VDD prevalence among HIV group, TB group, and HIV-TB group, as well as the effects of vitamin D supplementation on the HIV group, TB group, and HIV-TB group. Meta-analyses of individual participant data can identify factors that explain differences in outcomes across studies.

## 2 Methods

### 2.1 Study design

The methods for this systemic review and meta-analysis were described in an outline protocol that was registered with the PROSPERO International Prospective Register of Systematic Reviews (identifier CRD42023478013). The meta-analysis was performed and reported according to the Preferred Reporting Items for Systematic Reviews and Meta-Analysis (PRISMA) guidelines (21).

### 2.2 Data sources and searches

We conducted a comprehensive literature search in the PubMed, Web of Science databases, the Cochrane Library, and Embase. The medical subject headings and free terms adopted were as follows: vitamin D (vitamin D2, vitamin D3, cholecalciferol, ergocalciferol, alphacalcidol, calcitriol, paricalcitol and doxercalciferol), human immunodeficiency virus (HIV), acquired immune deficiency syndrome (AIDS), and tuberculosis (TB). The search was limited to English journal articles. The detailed search strategy is described in Supplementary Table S1. Studies that fulfilled both the inclusion and exclusion criteria published before November 1, 2023 were included.

### 2.3 Study selection

The inclusion criteria were as follows: (1) human study related to HIV, TB, or HIV-TB, (2) participants over 18 years of age diagnosed with HIV, TB, or HIV-TB infected, (3) double-blind, placebo-controlled RCTs of vitamin D supplementation, (4) observation study with a comparable HIV group, TB group, and HIV-TB group, (5) studies in which a factorial design was used to investigate effects of other therapies alongside vitamin D were included, as these allowed effects of vitamin D to be isolated.

Exclusion criteria included the following: (1) studies in which vitamin D was given in combination with another intervention were excluded if the effects of vitamin D could not be isolated (e.g., by use of a factorial design), (2) no control group for comparison or unclear information for the control group, (3) abstract and conference proceeding, short or brief communication, (4) case reports or case series, (5) basic experimental studies, (6) non-English language studies, (7) participants included pregnant women.

All the studies search from four databases were sent to citation manager (Endnote X9). After removing duplicates by using the citation manager, researchers (K.D.X. and Y.Z.) read via the titles and abstracts of the studies independently. We narrowed down the list in this way and then read the full text of the remaining articles. Full texts were obtained, and further screening was performed when the studies were recognized as eligible or uncertain with respect to their eligibility. Disagreements during the screening process were resolved by discussion with the third team member.

### 2.4 Data extraction

Two researchers (K.D.X. and Y.Z.) independently extracted and organized the data using an Excel spreadsheet. Disagreements were resolved by discussion. The following data were also extracted: study, setting, study design, latitude, HIV group (age, male proportion, BMI, CD4 T-cell counts), TB group (age, male proportion, BMI, CD4 T-cell counts), HIV-TB group (age, male proportion, BMI, and CD4 T-cell counts), method of vitamin D measurement, mean serum vitamin D level, vitamin D deficiency prevalence, dose of vitamin D (intervention arm), follow-up time after treatment initiation (months), HIV viral load, time to sputum smear conversion, time to culture conversion, relapse, morality, and TB score.

### 2.5 Risk of bias

Researchers assessed the quality of the included studies, cohort studies were evaluated using the Newcastle-Ottawa Scale. The cross-sectional studies were evaluated using a scale launched by the Agency for Healthcare Research and Quality. Selection and comparability were considered for both case–control and cohort studies. Moreover, exposure assessment was performed for case–control studies and outcome assessment was performed for cohort studies. The highest score is 9. A score <5 was considered high risk of bias, 5–7 was considered moderate risk of bias, and <7 was considered low risk of bias. A total of eleven items were judged in the cross-sectional study.

### 2.6 Definition of outcomes

The results of this review were mainly divided into primary outcomes and secondary outcomes. The primary outcome were differences in vitamin D level and VDD prevalence between HIV group, TB group, and HIV-TB. The secondary outcomes were CD4 count and viral load in the HIV group after vitamin D supplementation; positive/negativity culture status, time to sputum smear conversion, time to culture conversion, relapse, and death in the TB group after vitamin D supplementation; 12-month morality, TB score, and time to sputum smear conversion in the HIV-TB group after vitamin D supplementation.

### 2.7 Data synthesis

We expressed variables by their mean and standard deviations (SD). N represents the sample size or the number of participants. For two study that expressed outcome in medians and interquartile range (IQR) were converted to means and standard deviations using Luo et al.’s (22) and Wan et al.’s (23) approaches. Data presented only with medians were excluded from the final meta-analysis. Serum vitamin D levels were converted into nmol/L by multiplying by 2.5 when they were presented as ng/mL^2^.

### 2.8 Subgroup analysis

We analyzed predefined subgroup by latitude (>30 or ≤30) and CD4 count (>200 cells/mm^3^ or ≤200 cells/mm^3^) to determine the factors affecting heterogeneity.

### 2.9 Statistical analysis

Search results were sent to citation manager software. We performed statistical analysis on pooled means and standard deviations of serum vitamin D concentration using Review Manager. Heterogeneity was expected to be high due to the numerous factors affecting vitamin D synthesis, such as season, race, and latitude. Therefore, we used a random-effects model for data synthesis (24). Heterogeneity was tested using the *I*^2^ test, with <25% being low heterogeneity, 25 ~ 50% being moderate heterogeneity, >50% being high heterogeneity, and <75% being high heterogeneity (25). A significant difference was considered with a value of *p* < 0.05.

## 3 Results

### 3.1 Study characteristics

**Figure 1:**
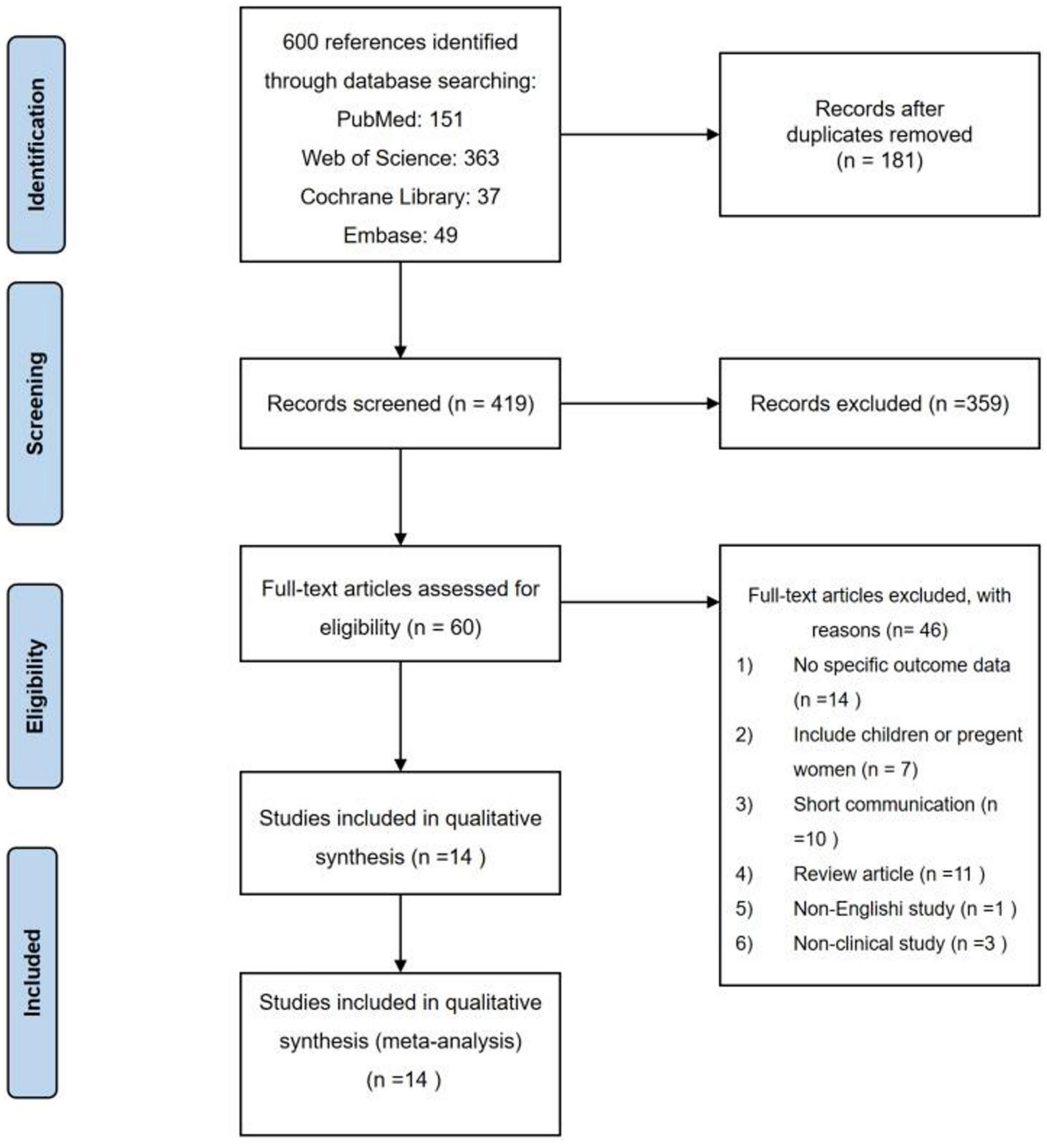
Flowchart of the study selection.

Among a total of 600 studies initially identified in the literature searching, 419 studies were further screened after removing duplicates. After screening the titles and abstracts, 60 studies were left for full-text assessment. Finally, 14 studies were included in our analysis. Details of the search progress are shown in Figure 1. Of the 14 studies, 3 were cohort studies (9, 26, 27), 5 were cross-sectional studies (28–32), and 6 were randomized double-blind placebo-controlled trials (33–38) (Tables 1–5). Two studies were performed in Uganda (26, 31), 8 in Africa (27–30, 35–38), and 3 in India (32–34). Furthermore, one study were performed in 9 countries (9), including Brazil, Haiti, India, Malawi, Peru, South Africa, Thailand, the United States, and Zimbabwe. Twenty-five (OH) D was detected by a RIA with ^125^I-labeled 25(OH)D [^125^I-25(OH)D] as tracer using a kit from IDS (Immunodiagnostic Systems) by Capio Diagnostics (28), ELISA-IDS (32), chemiluminescent assay (26), liquid chromatography–tandem mass spectrometry (LCMS/MS) (27, 29), liquid chromatography assay (31), DiaSorin (Stillwater, MN) (9), chemiluminescent immunoassay analyser (30). We included studies that defined vitamin D deficiency as serum 25(OH)D <50 nmol/L (26, 27, 29), serum 25(OH)D concentration <20 ng/mL (30, 32) or serum 25(OH)D concentration <12 ng/mL (31). The primary characteristics of the included studies are presented in Tables 1–5. The risk of bias from the included articles is shown in Tables 6, 7.

**Table 1:** The characteristics of included studies for assessing vitamin D level between HIV group and HIV-TB group.

Study	Setting	Latitude	Study design	HIV age (years)	HIV-TB age (years)	Sex, male *n* (%)	BMI	CD4 T-cell counts	No. of HIV	No. of HIV-TB	VD level in HIV	VD level in HIV-TB
Nansera et al. (31)	South-Western Uganda	0.6132 S	Cross-sectional study	35 ± 10^*^	37 ± 10^*^	HIV: 19 (38) HIV-TB: 29 (58)	HIV: 22.8 ± 3.8^*^ HIV-TB: 19.8 ± 3.8^*^	HIV: 372 ± 256^*^ HIV-TB: 213 ± 151^*^	50	50	28 ± 11^*^ ng/ml	24 ± 11^*^ ng/ml
Conesa-Botella et al. (26)	South-Western Uganda	33°55′S	Cohort study	34 (29–40)^#^	34 (28–39)^#^	HIV: 12 (60) HIV-TB: 52 (56)	NA	HIV: 27 (17–69) ^#^ HIV-TB: 25.5 (14–74)^#^	20	92	79 (64–102)^#^ nmol/L	81 (61–105)^#^ nmol/L
Musarurwa et al. (30)	Harare, Zimbabwe, Africa	17°55′S	Cross-sectional study	40.5 (12.6)^*^	38.6 (9.0)^*^	HIV: 70 (50) HIV-TB: 63 (48)	NA	NA	139	145	20.4 (14.6–26.9)^#^ ng/ml	25.3 (18.0–33.7)^#^ ng/ml
Tenforde et al. (9)	Brazil (10%), Haiti (10%), India (14.8%), Malawi (12.3%), Peru (9.3%), South Africa (14.2%), Thailand (9.6%), the United States (9.6%), Zimbabwe (10.2%)	NA	Cohort study	35 (29–41)^#^	34 (29–39)^#^	HIV: 129 (51) HIV-TB: 46 (60)	HIV: 22.3 (20.1–25.1)^#^ HIV-TB: 21.6 (19.5–22.9)^#^	HIV: 180 (90–231)^#^ HIV-TB: 136 (60–201)^#^	255	77	32 (24–39)^#^ ng/ml	30 (21–35)^#^ ng/ml

^*^, Mean ± SD; ^#^, Median [IQR]; NA, not available; ND, not determined.

**Table 2:** The characteristics of included studies for assessing vitamin D level between TB group and HIV-TB group.

Study	Setting	Latitude	Study design	TB age (years)	HIV-TB age (years)	Sex, male *n* (%)	BMI	CD4 T-cell counts	No. of TB	No. of HIV-TB	VD level in TB	VD level in HIV-TB
Friis et al. (28)	Tanzania, East Africa	2.28 S	Cross-sectional study	NA	NA	NA	NA	NA	344	309	86.3 (82.8–89.9)^#^ nmol/L	86.9 (83.3–90.6)^#^ nmol/L
Martineau et al. (29)	South Africa	33°S	Cross-sectional study	31.7 (25.8–42.3)^#^	32.0 (27.0–38.2)^#^	TB: 64 (68.8) HIV-TB: 40 (40.4)	TB: 20.1 (18.1–22.5)^#^ HIV-TB: 21.1 (19.0–24.1)^#^	TB: ND HIV-TB: 167 (57–292)^#^	93	99	40.5 (20.8)^*^ nmol/L	28.7 (19.1)^*^ nmol/L
Conesa-Botella et al. (26)	South-Western Uganda	33°55′S	Cohort study	25 (22–30)^#^	34 (28–39)^#^	TB: 14 (51.8) HIV-TB: 52 (56)	NA	TB: ND HIV-TB: 25.5 (14–74)^#^	27	92	78 (62–92)^#^ nmol/L	81 (61–105)^#^ nmol/L
Musarurwa et al. (30)	Harare, Zimbabwe, Africa	17°55′S	Cross-sectional study	39.7 (16.5)^*^	38.6 (9.0)^*^	TB: 84 (64) HIV-TB: 63 (48)	NA	NA	134	145	24.0 (19.5–29.6)^#^ ng/ml	25.3 (18.0–33.7)^#^ ng/ml

^*^, Mean ± SD; ^#^, Median [IQR]; NA, not available; ND, not determined.

**Table 3:** The characteristics of included studies for assessing vitamin D deficiency prevalence between HIV group and HIV-TB group.

Study	Setting	Latitude	Study design	HIV age (years)	HIV-TB age (years)	Sex, male *n* (%)	BMI	CD4 T-cell counts	No. of HIV	No. of HIV-TB	No. of VDD of HIV cohort	No. of VDD of HIV-TB cohort
Nansera et al. (31)	Mwanza, Tanzania, East Africa	0.6132 S	Cross-sectional study	35 ± 10^*^	37 ± 10^*^	HIV: 19 (38) HIV-TB: 29 (58)	HIV: 22.8 ± 3.8^*^ HIV-TB:19.8 ± 3.8^*^	HIV: 372 ± 256^*^ HIV-TB: 213 ± 151^*^	50	50	5	6
Conesa-Botella et al. (26)	South Africa	33°55′S	Cross-sectional study	34 (29–40)^#^	34 (28–39)^#^	HIV: 12 (60) HIV-TB: 52 (56)	NA	HIV: 27 (17–69)^#^ HIV-TB: 25.5 (14–74)^#^	20	92	3	15
Musarurwa et al. (30)	South-Western Uganda	17°55′S	Cohort study	40.5 (12.6)^*^	38.6 (9.0)^*^	HIV: 70 (50) HIV-TB: 63 (48)	NA	NA	139	145	67	49
Sinha et al. (32)	North India	NA	Cross-sectional study	33 ± 11^*^	35 ± 10^*^	HIV: 36 (75) HIV-TB: 17 (79)	HIV: 22.4 ± 3.6^*^ HIV-TB: 22.3 ± 3.7^*^	HIV: 292 (8–915)^#^ HIV-TB: 234 (0–669)^#^	48	24	13	5

^*^, Mean ± SD; ^#^, Median [IQR]; NA, not available; ND, not determined.

**Table 4:** The characteristics of included studies for assessing vitamin D deficiency prevalence between TB group and HIV-TB group.

Study	Setting	Latitude	Study design	TB age (years)	HIV-TB age (years)	Sex, male *n* (%)	BMI	CD4 T-cell counts	No. of TB	No. of HIV-TB	No. of VDD of TB cohort	No. of VDD of HIV-TB cohort
Martineau et al. (29)	South Africa	33°S	Cross-sectional study	31.7 (25.8–42.3)^#^	32.0 (27.0–38.2)^#^	TB: 64 (68.8) HIV-TB: 40 (40.4)	TB: 20.1 (18.1–22.5)^#^ HIV-TB: 21.1 (19.0–24.1)^#^	TB: ND HIV-TB: 167 (57–292)^#^	93	99	70	85
Conesa-Botella et al. (26)	South Africa	33°55′S	Cross-sectional study	25 (22–30)^#^	34 (28–39)^#^	TB: 14 (51.8) HIV-TB: 52 (56)	NA	TB: ND HIV-TB: 25.5 (14–74)^#^	27	92	4	15
Mehta et al. (27)	Tanzania, East Africa	NA	Cohort study	30.2 ± 9.2^*^	34.3 ± 8.6^*^	TB: 257 (77.2) HIV-TB: 203 (59.0)	TB:18.8 ± 2.5^*^ HIV-TB:19.4 ± 2.8^*^	TB: 709.2 ± 250.8^*^ HIV-TB: 327.2 ± 246.2^*^	333	344	51	55
Musarurwa et al. (30)	South-Western Uganda	17°55′S	Cohort study	39.7 (16.5)^*^	38.6 (9.0)^*^	TB: 84 (64) HIV-TB: 63 (48)	NA	NA	134	145	38	49
Sinha et al. (32)	North India	NA	Cross-sectional study	42 ± 13^*^	35 ± 10^*^	TB: 22 (62) HIV-TB: 17 (79)	TB: 21.6 ± 4.3^*^ HIV-TB: 22.3 ± 3.7^*^	TB: ND HIV-TB: 234 (0–669)^#^	37	24	14	5

^*^, Mean ± SD; ^#^, Median [IQR]; NA, not available; ND, not determined.

**Table 5:** The characteristics of included studies for assessing effects of vitamin D supplementation on mortality in HIV-TB group.

Study	Setting	Age (years)	Sex (male %)	Study design	Anti-HIV therapy	Anti-TB therapy	Baseline 25(OH)D	Dose of vitamin D: intervention arm	Follow-up time after treatment initiation (months)
Wejse et al. (38)	Guinea-Bissau, West Africa	37 (13)^#^,^*^	116 (62%)^*^	Randomized, double-blind, placebo-controlled trial	NR	2 months HRZE, then 6 months HE	77.5 (23.8) ^*^ nmol/L	100,000 IU of cholecalciferol at inclusion and again 5 and 8 months after the start of treatment	visit 12 months after initiation of treatment, or until death or moving out of the study area
Sudfeld et al. (36)	Tanzani, East Africa	38.6 (9.8)^#^	634 (32%)	Randomized, double-blind, placebo-controlled trial	Efavirenz–lamivudine–tenofovir	2 months HRZE, then 4 months HR	<30 ng/mL	50,000 IU at randomization and once a week for 3 weeks at clinic visits and 2000 IU at the fourth week until trial discharge at 1 year post ART initiation	at baseline, 1, 6, and 12 months

NR, not report; ^#^, mean (SD); ^*^% of all participants; H, isoniazid; R, rifampicin; Z, pyrazinamide; E, ethambutol.

**Table 6:** Quality assessment of included studies.

References	Study type	Selection				Comparability	Exposure/Outcome			Final score
		Item 1	Item 2	Item 3	Item 4	Item 5	Item 6	Item 7	Item 8	
Conesa-Botella et al. (26)	Cohort study	1	1	1	0	1	0	1	0	5
Mehta et al. (27)	Cohort study	1	1	1	0	1	0	1	0	5
Tenforde et al. (9)	Cohort study	1	1	1	0	2	0	1	0	6

For cohort study: Item 1, Representativeness of the exposed cohort; Item 2, Selection of the non-exposed cohort; Item 3, Ascertainment of exposure; Item 4, Demonstration that outcome of interest was not present at start of study; Item 5, Comparability of cohorts on the basis of the design or analysis; Item 6, Assessment of outcome; Item 7, Was follow-up long enough for outcomes to occur; Item 8, Adequacy of follow up of cohorts.

**Table 7:** Quality assessment of included cross-sectional studies.

References	Item 1	Item 2	Item 3	Item 4	Item 5	Item 6	Item 7	Item 8	Item 9	Item 10	Quality
Friis et al. (28)	Yes	Yes	Yes	Unclear	No	Yes	No	Yes	Unclear	Yes	Moderate
Martineau et al. (29)	Yes	No	Yes	Unclear	No	Yes	No	Yes	Unclear	Yes	Moderate
Nansera et al. (31)	Yes	No	Yes	Unclear	No	Yes	No	Yes	Unclear	No	Moderate
Musarurwa et al. (30)	Yes	Yes	Yes	Unclear	No	Yes	No	Yes	Unclear	No	Moderate
Sinha et al. (32)	Yes	Yes	Yes	Unclear	No	Yes	No	Yes	Unclear	Yes	Moderate

The 10 evaluation items are as follows: Item 1, Define the source of information (survey, record review); Item 2, List the inclusion and exclusion criteria for exposed and unexposed subjects (cases and controls) or refer to previous publications; Item 3, Indicate the time period used for identifying patients; Item 4, Indicate whether or not the subjects were consecutive, if not population-based; Item 5, Indicate if the evaluators of the subjective components of the study were masked to other aspects of the status of the participants; Item 6, Describe any assessments undertaken for quality assurance purposes (e.g., test/retest of primary outcome measurements); Item 7, Explain the exclusion of any patient from the analysis; Item 8, Describe how confounding variables were assessed and/or controlled; Item 9, If applicable, explain how missing data were handled in the analysis; Item 10, Summarize the patient response rates and completeness of the data collection.

### 3.2 Comparison of vitamin D levels in HIV group, TB group, and HIV-TB group

#### 3.2.1 Differences in vitamin D levels between HIV group and TB group

**Figure 2:**
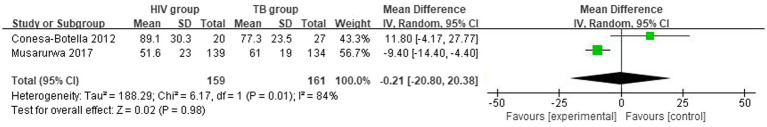
Vitamin D levels in HIV group and TB group.

The mean and SD of serum 25(OH)D level reported by 2 studies were pooled and computed. There were no statistically significant differences in vitamin D level between HIV group and TB group. The overall mean difference (MD) between HIV group and TB group was −0.21 (95% CI, −20.80 to 20.38; *p* = 0.98; *I*^2^ = 84%). The forest plots are shown in Figure 2. Because of the lack of studies, we could not determine the origin of heterogeneity.

#### 3.2.2 Differences in vitamin D levels between HIV group and HIV-TB group

**Figure 3:**
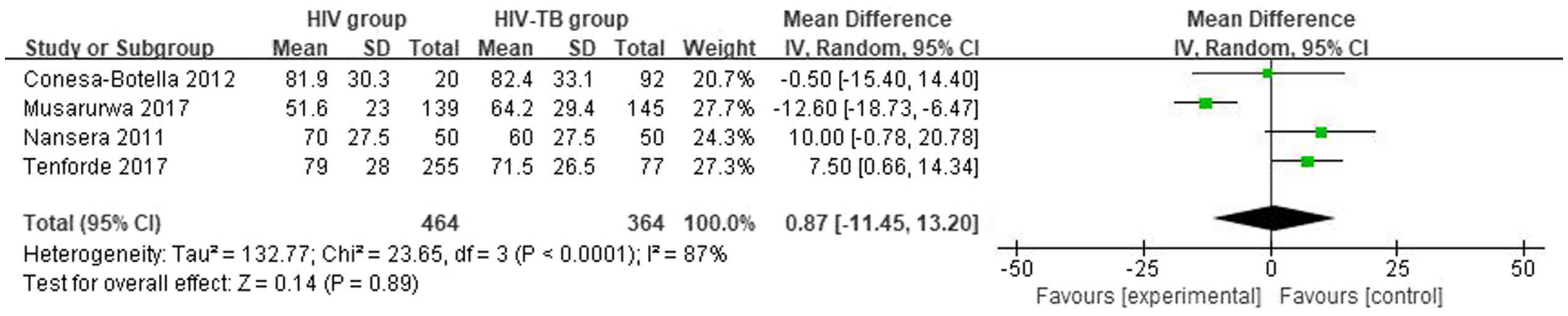
Vitamin D levels in HIV group and HIV-TB group.

The mean and SD of serum 25(OH)D level reported by 4 studies were pooled and computed. There were no statistically significant differences in vitamin D level between HIV group and HIV-TB group. The overall MD between HIV group and HIV-TB group was 0.87 (95% CI, −11.45 to 13.20; *p* = 0.89; *I*^2^ = 87%). The forest plots are shown in Figure 3. In subgroup analyses, when we grouped the CD4 count less than or equal to 200 cells/mm^3^ patients in the study with the CD4 count more than 200 cells/mm^3^ patients (39), the overall MD was 7.08 (95% CI, 1.69–12.47; *p* = 0.01; *I*^2^ = 0%; Supplementary Figure S1) from 3 studies. Moreover, we grouped the latitude less than or equal to 30 with the latitude more than 30, the overall MD was −1.54 (95% CI, −16.85–13.76; *p* = 0.84; *I*^2^ = 85%; Supplementary Figure S2) from 3 studies.

#### 3.2.3 Differences in vitamin D levels between TB group and HIV-TB group

**Figure 4:**
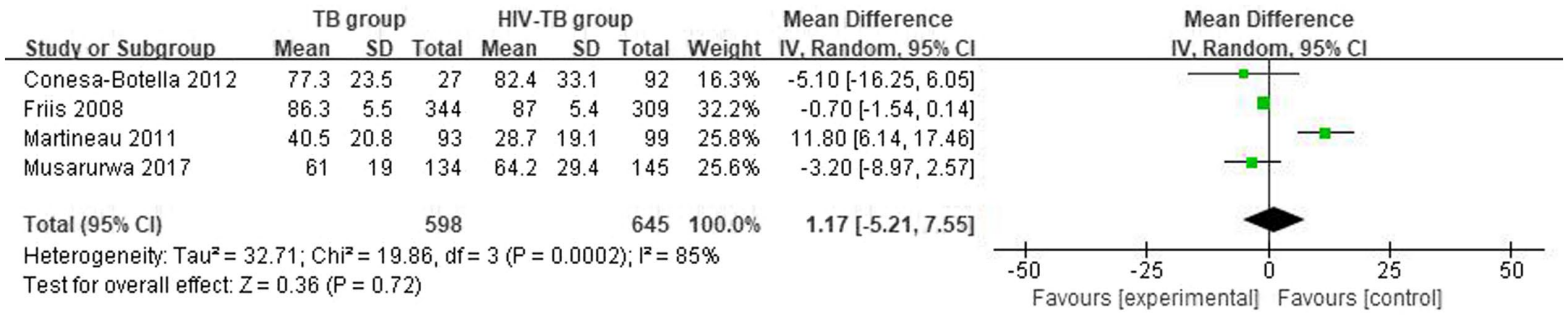
Vitamin D levels in TB group and HIV-TB group.

The mean and SD of serum 25(OH)D level reported by 4 studies were pooled and computed. There were no statistically significant differences in vitamin D level between TB group and HIV-TB group. The overall MD between TB group and HIV-TB group was 1.17 (95% CI, −5.21–7.55; *p* = 0.72; *I*^2^ = 85%). The forest plots are shown in Figure 4. In subgroup analyses, we grouped the latitude less than or equal to 30 with the latitude more than 30, the overall MD was 1.17 (95% CI, −5.21–7.55; *p* = 0.72; *I*^2^ = 85%; Supplementary Figure S3) from 3 studies.

### 3.3 Comparison of vitamin D deficiency prevalence in HIV group, TB group, and HIV-TB group

#### 3.3.1 Differences in the vitamin D deficiency prevalence between HIV group and TB group

**Figure 5:**
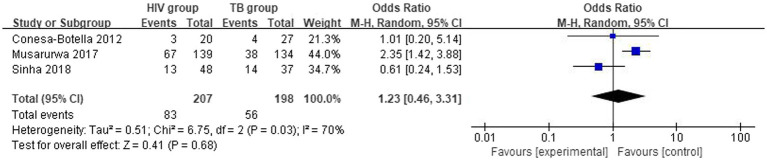
Vitamin D deficiency prevalence in HIV group and TB group.

The overall number of VDD events and sample size of vitamin D group and the placebo group from 3 studies were combined and calculated. There were no statistically significant differences in VDD prevalence between HIV group and TB group. The overall odds ratio (OR) for HIV group vs. the TB group was 1.23 (95% CI, 0.46–3.31; *p* = 0.68; *I*^2^ = 70%). The forest plots are shown in Figure 5. Because of the lack of studies and data, we could not determine the origin of heterogeneity.

#### 3.3.2 Differences in the vitamin D deficiency prevalence between HIV group and HIV-TB group

**Figure 6:**
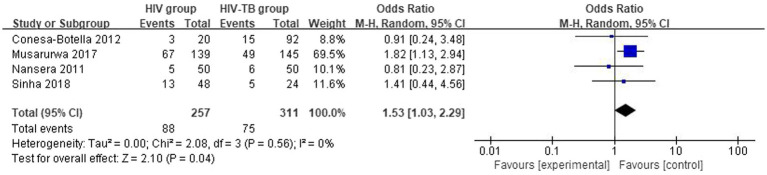
Vitamin D deficiency prevalence in HIV group and HIV-TB group.

The overall number of VDD events and sample size of vitamin D group and the placebo group from 4 studies were combined and calculated. There were differences in VDD prevalence between HIV group and HIV-TB group. The overall OR for HIV group vs. the HIV-TB group was 1.53 (95% CI, 1.03–2.29; *p* = 0.04; *I*^2^ = 0%). The forest plots are shown in Figure 6. HIV-TB group were more susceptible to VDD than HIV group.

#### 3.3.3 Differences in the vitamin D deficiency prevalence between TB group and HIV-TB group

**Figure 7:**
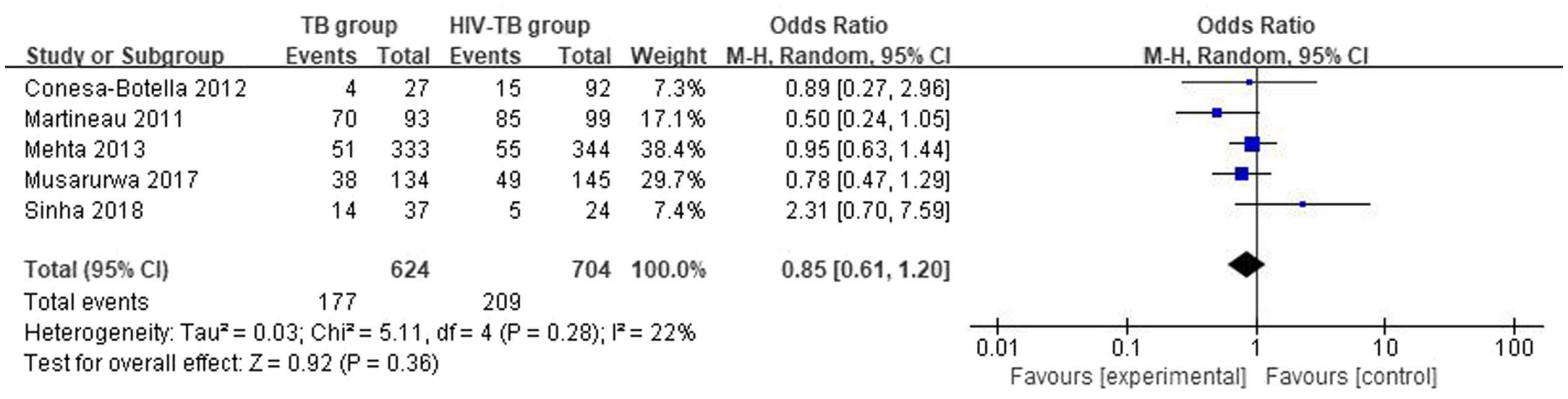
Vitamin D deficiency prevalence in TB group and HIV-TB group.

The overall number of VDD events and sample size of vitamin D group and the placebo group from 5 studies were combined and calculated. There were no statistically significant differences in VDD prevalence between TB group and HIV-TB group. The overall OR for TB group versus the HIV-TB group was 0.85 (95% CI, 0.61–1.20; *p* = 0.36; *I*^2^ = 22%). The forest plots are shown in Figure 7.

### 3.4 Effects of vitamin D supplementation on HIV group, TB group, and HIV-TB group

#### 3.4.1 Effects of vitamin D supplementation on HIV group

Steenhoff et al. explored the effects of vitamin D_3_ in HIV group for different durations (baseline, 6 weeks, and 12 weeks) by supplementing with different doses (4,000 IU or 7,000 IU) of vitamin D. This led to the finding that supplementation with a high dose of vitamin D for 12 weeks is safe and can improve HIV status (CD4 count increased and viral load decreased) (35).

#### 3.4.2 Effects of vitamin D supplementation on TB group

Daley et al. used vitamin D_3_ intervention (four doses of 2.5 mg at weeks 0, 2, 4, and 6) or placebo for pulmonary tuberculosis (PTB) patients, found that the proportion of sputum culture negativity at day 56 did not differ significantly and was similar between groups. Furthermore, they detected that median time to culture conversion did not differ significantly between patients in the vitamin D group and those in the placebo group, and noted no significant difference between groups in time to culture conversion and time to smear conversion (33). Likewise, Wallis et al. utilized ergocalciferol (5 mg on day 1, then 2.5 mg on day 28 and day 56) intervention or standard treatment alone (the control group) for PTB patients, discovered that the two treatments had no significant effect on positive culture status at 56 days or on the hazard ratio for stable culture conversion up to day 180 (37). Sinha et al. exploited vitamin D_3_ intervention (60,000 IU/sachet weekly for first 2 months, fortnightly for next 4 months followed by monthly for the next 18 months) or placebo in patients with PTB, found there is no significant difference in the time to sputum smear conversion between the two groups (*p* = 0.358) and no significant difference in the time to culture conversion between the two groups (*p* = 0.418) (34). In addition, Sinha et al. further observed the relapse of PTB and obtained the result that there was not statistically significant between the two groups (*p* = 0.29). For mortality (all-cause deaths), the data from Sinha et al. showed no death was directly attributable to the study intervention (34).

#### 3.4.3 Effects of vitamin D supplementation on HIV-TB group

**Figure 8:**
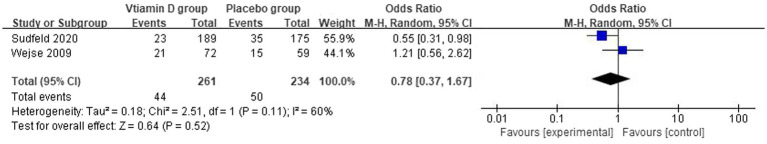
Mortality with vitamin D supplementation or placebo among HIV-TB co-infected patients.

The overall number of events of mortality and sample size of vitamin D group and the placebo group from 2 studies were combined and calculated. Vitamin D supplementation did not have a statistically significant effect on the mortality of HIV/TB co-infected patients. The overall OR for vitamin D group vs. the placebo group was 0.78 (95% CI, 0.34–1.67; *p* = 0.52; *I*^2^ = 60%). The forest plots are shown in Figure 8. Furthermore, Wejse et al. reported that vitamin D does not improve clinical outcome among patients with TB (38). Because of the lack of studies, we could not determine the origin of heterogeneity.

## 4 Discussion

We reported results of the meta-analysis of participant data from cohort studies and cross-sectional studies of vitamin D levels in HIV-infected patients, TB-infected patients, and HIV-TB co-infected patients, and from RCTs of supplement vitamin D in HIV-infected patients, TB-infected patients, and HIV-TB co-infected patients. The review in the first to quantitatively and systematically compare serum vitamin D levels in the three group of patients (HIV group, TB group, and HIV-TB group) and VDD prevalence. Moreover, we also explored the effect of vitamin D supplementation on three groups of people. Likewise, there was no significant difference in vitamin D levels between the HIV group, TB group, and HIV-TB group. However, the prevalence of vitamin D deficiency was higher in the HIV-TB group than in the HIV group.

Our overall finding of a higher vitamin D deficiency prevalence tended to occur in HIV-TB infected patients is consistent with results from existing systematic reviews and aggregate data meta-analyses. Some mechanisms have been explored to interpret the relationship between HIV, HIV-TB and VDD. HIV-TB co-infected patients have many more risk factors than HIV infected patients (9, 40). The influence of traditional factors involving latitude, BMI, and age is apparent in HIV-infected patients (3). Furthermore, the main predictors of VDD in patients with TB are lack of UV exposure, inadequate dietary intake, comorbidities, and old age (8). The initial step of vitamin D metabolism occurs in the skin, in which sunlight plays an important role. Adequate sunlight is easy to obtain in lower latitude places. At the same time many of the studies we included were conducted in Africa.

In the double-blind randomized controlled studies of vitamin D supplementation that we included, there was no significant improvement in either CD4 counts and viral load in the HIV group population. Vitamin D supplementation also had no significant effect on time to sputum smear conversion, time to culture conversion, relapse, and death in TB-infected patients compared with the placebo group. These outcomes may be related to insufficient doses of vitamin D supplements. More researches are needed before implementation of vitamin D supplementation in HIV care and treatment programs to prevent tuberculosis or death is considered. Likewise, vitamin D have no apparent benefit on mortality in HIV-TB co-infected patients, nor on clinical symptoms of tuberculosis. By analyzing the two studies separately, we could see that the vitamin dose used by Wejse et al. (38) is 100,000 IU of cholecalciferol at inclusion and again 5 and 8 months after the start of treatment. The conclusion during the 12-month follow-up was that vitamin D supplementation can effectively reduce the mortality rate in the susceptible population, while the vitamin D used by Sudfeld et al. (36) was 50,000 IU at randomization and once a week for 3 weeks at clinic visits and 2000 IU at the fourth week until trial discharge at 1 year post ART initiation, the conclusion reached after a 12-month follow-up is that vitamin D supplementation has no effect on mortality in co-infected people. Therefore, we considered that the reason for the difference in the conclusions of these two studies may be the different dose of vitamin D supplementation.

## 5 Limitations

Several points should be considered in interpreting our results. Frist, we only adopted the baseline data into our meta-analysis from all the literature. We could not obtain a causal association from these studies because the change in vitamin D may interfere with lifestyle. Second, we could not obtain enough RCTs of vitamin D supplementation in HIV/TB co-infected patients. Third, our included studies were limited to the English language and adult. In our review, the included studies were conducted only in several countries, it is unclear whether this association exists elsewhere. Finally, age, sex, BMI, latitude, type of HIV patients, TB patients, HIV/TB co-infected patients, CD4 count, ART, and anti-TB treatment could be sources of heterogeneity. Due to the limited number of studies involved in each outcome and insufficient data, we implemented subgroup analyses whenever possible. However, we adopted random-effect model and performed subgroup analysis to reduce the effect of heterogeneity as much as possible. We analyzed predefined subgroup by latitude (>30 or ≤ 30) and CD4 count (>200 cells/mm^3^ or ≤ 200 cells/mm^3^) to determine the factors affecting heterogeneity. Mikua et al. found that vitamin D levels were positively correlated with CD4 percentage in HIV-infected patients (*r* = 0.17, *p* = 0.036) (41). In contrast, Flauzino et al. found no significant differences in CD4 count (categorized into <200, 200–500, and > 500 groups) when stratified by vitamin D levels (vitamin D < 30 ng/mL vs. ≥30 ng/mL) (*p* = 0.426) (42). We found a significant association between the two outcomes by subgrouping according to CD4 count. HIV group and HIV-TB group in the studies with a CD4 count of less than 200 cells/mm^3^ were found to have lower vitamin D levels than with a CD4 count of more than 200 cells/mm^3^. Furthermore, wang et al. found HIV group has a high risk of VDD at lower latitude (3). However, our subgroup analysis according to latitude showed that the results did not change significantly.

## 6 Conclusion and future directions

In summary, our findings indicated that there were no variations in vitamin D levels between HIV infected individuals, TB infected individuals, and HIV-TB co-infected individuals. The prevalence of vitamin D deficiency was higher in the HIV-TB group than in the HIV group. Additionally, the administration of vitamin D supplements did not have obvious impact on CD4 count and viral load in the HIV group. Likewise, vitamin D had no effect on time to sputum smear conversion, time to culture conversion, relapse, and death in the TB group. Vitamin D supplementation had no effect on 12-month morality and TB score in the HIV-TB group. The appropriate dose of vitamin D supplementation for HIV-infected patients, TB-infected patients, and HIV-TB co-infected patients is unclear, and the amount of vitamin D supplementation varied in the study, which affected the results of the trial. Therefore, additional randomized, controlled studies must be conducted to explore the appropriate and safe dosage of vitamin D supplementation in order effectively improve the progression of HIV infected individuals, TB infected individuals, and HIV-TB co-infected individuals.

## Data availability statement

The original contributions presented in the study are included in the article/Supplementary material, further inquiries can be directed to the corresponding authors.

## Author contributions

KX: Writing – original draft, Writing – review & editing. YZ: Writing – original draft, Writing – review & editing. MZ: Writing – original draft, Writing – review & editing. HW: Supervision, Writing – original draft, Writing – review & editing. LZ: Writing – original draft, Writing – review & editing. JJ: Writing – original draft, Writing – review & editing. ZL: Supervision, Writing – original draft, Writing – review & editing. WW: Supervision, Writing – original draft, Writing – review & editing. TZ: Supervision, Writing – original draft, Writing – review & editing.
