# Supplementary material for: Association of vitamin D with HIV infected individuals, TB infected individuals, and HIV-TB co-infected individuals: a systematic review and meta-analysis
Source: Front Public Health. 2024 Feb 14;12:1344024. doi: 10.3389/fpubh.2024.1344024 (PMC10910524; doi:10.3389/fpubh.2024.1344024)
Supplement: Supplementary file 1 [file Data_Sheet_1.docx]

**Association of vitamin D with HIV infected individuals, TB infected individuals, and HIV-TB co-infected individuals: A Systematic Review and Meta-Analysis**

**Search Strategies**

1. PubMed

***Terms specific to vitamin D***

#1. Vitamin D OR vitamin D2 OR vitamin D3 OR cholecalciferol OR ergocalciferol OR alphacalcidol OR alfacalcidol OR calcitriol OR paricalcitol OR doxercalciferol

***Terms specific to tuberculosis***

#2. Tuberculosis

***Terms specific to human immunodeficiency virus***

#3. Human immunodeficiency virus OR HIV OR Acquired immune deficiency syndrome OR AIDS

***Combination of terms to identify the final results***

#1 AND #2 AND #3

1. Web of science

TS =(Vitamin D OR vitamin D2 OR vitamin D3 OR cholecalciferol OR ergocalciferol OR alphacalcidol OR alfacalcidol OR calcitriol OR paricalcitol OR doxercalciferol) AND TS =(Tuberculosis) AND TS=(Human immunodeficiency virus OR HIV OR Acquired immune deficiency syndrome OR AIDS)

1. Cochrane Library

***Terms specific to vitamin D***

#1. Vitamin D OR vitamin D2 OR vitamin D3 OR cholecalciferol OR ergocalciferol OR alphacalcidol OR alfacalcidol OR calcitriol OR paricalcitol OR doxercalciferol

***Terms specific to tuberculosis***

#2. Tuberculosis

***Terms specific to human immunodeficiency virus***

#3. Human immunodeficiency virus OR HIV OR Acquired immune deficiency syndrome OR AIDS

***Combination of terms to identify the final results***

#1 AND #2 AND #3

1. Embase

***Terms specific to vitamin D***

#1. Vitamin D OR vitamin D2 OR vitamin D3 OR cholecalciferol OR ergocalciferol OR alphacalcidol OR alfacalcidol OR calcitriol OR paricalcitol OR doxercalciferol

***Terms specific to tuberculosis***

#2. Tuberculosis

***Terms specific to human immunodeficiency virus***

#3. Human immunodeficiency virus OR HIV OR Acquired immune deficiency syndrome OR AIDS

***Combination of terms to identify the final results***

#1 AND #2 AND #3
