# Supplementary figures and images for: Association of vitamin D with HIV infected individuals, TB infected individuals, and HIV-TB co-infected individuals: a systematic review and meta-analysis
Source: Front Public Health. 2024 Feb 14;12:1344024. doi: 10.3389/fpubh.2024.1344024 (PMC10910524; doi:10.3389/fpubh.2024.1344024)

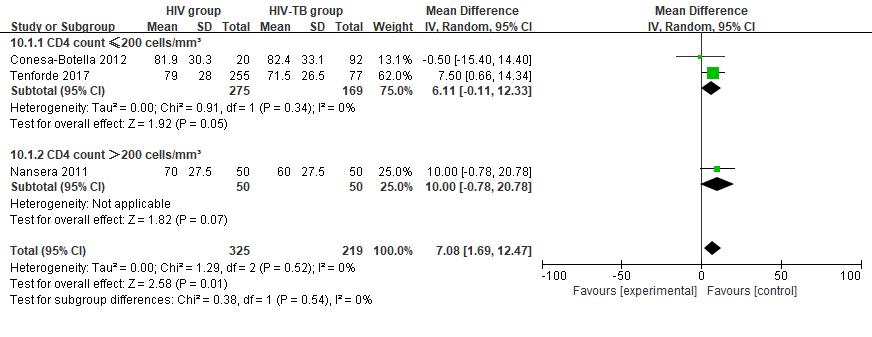

Supplement: Supplementary file 2 [file Image_1.JPEG]

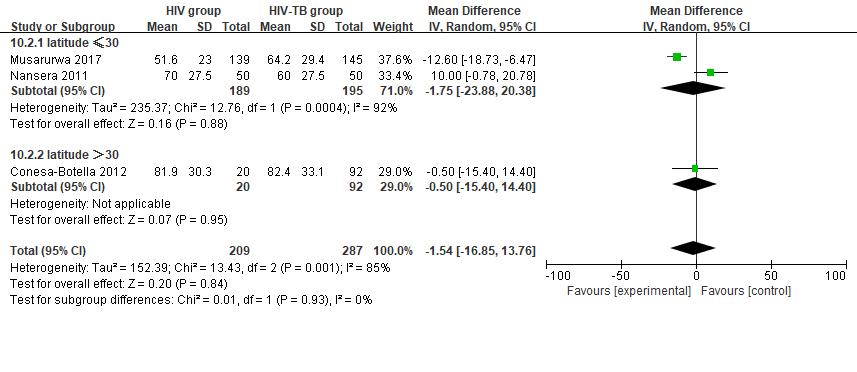

Supplement: Supplementary file 3 [file Image_2.JPEG]

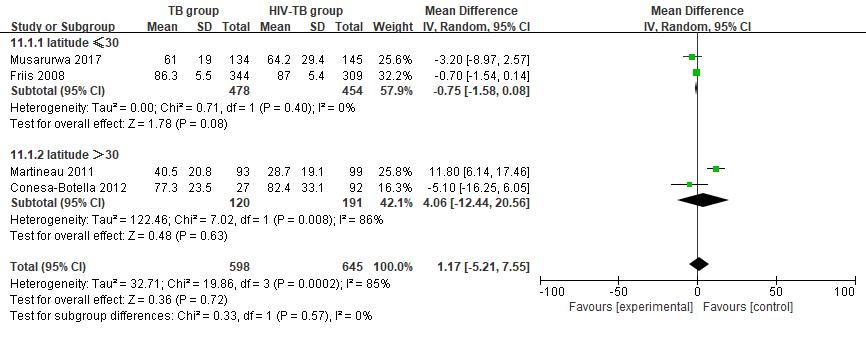

Supplement: Supplementary file 4 [file Image_3.JPEG]
